# United States Exploring Expedition, during the Years 1838, 1839, 1840, 1841, 1842, under the Command of Charles Wilkes, U. S. N. Vol. IX

**Published:** 1851-03

**Authors:** 


ART. 2.—*United States Exploring Expedition, during the years* 1838, 1839, 1840, 1841, 1842, *under the command of Charles Wilkes, U. S. N.* Vol. ix.

*The Races of Man, and their Geographical Distribution.* *By* CHAS. PICKERING, M. D., member of the scientific corps attached to the Expedition. Boston: Charles C. Little & James Brown.

*The Races of Men: A Fragment.* By ROBERT KNOX, M. D., Lecturer on Anatomy, and corresponding member of the National Academy of Medicine of France. Philadelphia: Lea & Blanchard.

*The New Englander*—1850.

*The North American Review*—1850.

*The Doctrine of the Unity of the Human Race examined on the principles of Science.* By JOHN BACHMAN, M. D., Professor of Natural History, College of Charleston, S. C.

There are few questions presented to the investigation of the explorer of nature, of greater importance than those comprised within the science of Ethnology. The profound depths of that science contain intellectual treasures of the highest value, and the farther the subject is penetrated the more we feel its value. The origin and destiny of the human race have ever been themes of momentous importance, and will continue to be until "corruption has put on incorruption." All philosophy, all truth, all of earthly interest, have these questions for their nucleus. The loftiest intellect known to pagan philosophy struggled first to find the KNOWN, and from that to reach after the unknown. Those who may freight their memories with Mr. Grote's full, perfect, and massive sketch of Socrates, will be at no loss to find how much of the labor of that high intelligence was devoted to the origin and destiny of the human family. And now, after two thousand years have rolled their terms since the Athenians heard philosophy from the voice of their greatest intellect, the question of the origin of man, is yet a matter of debate. Between the supposed conflicting elements of science and revelation, the subject has reached a state of entanglement that might have gratified Berkely, of Cloyne, Hobbes, or Hume. Science is widening its domains, and bringing many wholesome and vital truths home to the interests of every day life, but there are many who feel alarm at the corresponding fact, that efforts are constantly made to narrow down the possessions of Revelation, until what is left is scarcely worth retaining. Moses is a myth, his statements preposterous, and his institutions are denounced as being in the very spirit of their age, by persons who can find no antiquity equal to them. The loftiest inspirations of the Hebrew prophets are referred to as the distempered ravings of Jewish rhapsodists, and the Christian Dispensation is made to hang upon a reference to it by Josephus, by men who unhesitatingly credit Thucydides, whose history makes no mention of Socrates, who was the most notable man of the period, pourtrayed by the historian.

In the investigation of the origin of man, it is impossible to proceed without reference to the Bible. The question of the origin of the human race is indissolubly connected with its destiny, and if the origin of man is to be decided by science without the aid of revelation, what is to be the fate of the more important branch of the subject—the destiny of man? What does, what can science propose to do with that?

We do not design to enter upon an elaboration of the subject of Ethnology at present, but shall be content to submit a summary of the present position of the investigation. The North American Review, for January, 1851, in a notice of "the tendency of modern science," says, rather flippantly: "last year, when Agassiz announced the discovery of his science, that the races of men could not have been derived from one earthly parentage, a feeble outcry from very feeble voices rose, or strove to rise upon the air; but there came back no echo from public opinion, and it has already died away." Can it be that the North American Review has been the victim of anæsthesia? Are the voices of Dr. Bachman, and of the Reviewer in the New Englander of Professor Agassiz' doctrine, what the North American Review ranks among the feeble things of this world? Has there indeed been no echo from public opinion? The Review says "*it* has already died away," and we are at a loss to know what he means; we know not whether he tries to say the public opinion which gave no echo has already died away, or that the echo which never had an existence, has died.

No one can hold the merits, the high and ennobling qualities of Professor Agassiz in higher estimation than we do. In his departments of natural science, he has, perhaps, no equal, certainly no superior. We have given his prelections on the origin of man, all the careful, unbiassed study we were able to command, and without any response from his opponents we felt the conviction that he had failed to establish his doctrine. The necessity he feels for a separation of the questions, the unity and the origin of the race, shows the constraint that bound his intellect. The plurality of origins was not, as the N. A. Review says it was, a discovery of Agassiz' science, for it had been promulged long before geology came to light, and long before the discovery of specific fauna and flora, the basis on which Professor Agassiz builds his superstructure. The representative species of particuliar localities is insisted upon by Professor Agassiz, and he handles the argument with boldness and power. But as a man is a cosmopolite, in the largest sense in which that term can be applied to a living being, the analogical bearings derived from representative species of fauna and flora are not very perceptible.

The errors of chronologers have done much towards the creation of false issues on geological and ethnological subjects. A great deal is assumed, we think, when an attempt is made to establish any certain and indubitable basis for the age of the world upon the Mosaic records. The origin of man is developed, without any attempt to fix the time of that origin. That the human race has been on the globe a much longer time than chronologers assume in their calculations, we think susceptible of a great deal of proof. That there is an inconsistency in the pro gress of civilization which all human records and all human experience show to be very slow; that stage of civilization clearly and abundantly proved to be in existence, according to the earliest human records; and the age of humanity, assumed by chronologers, must strike the attention of all who investigate the subject. For instance, how can we account for the culmination of Assyrian civilization, if chronology be infallible, by any thing we know of the progress of any nation since Christianity dawned upon the world? The conceptions formed by readers of history, of the height reached by the Assyrian empire, were of a lofty character, but their loftiest reaches did not approach the developments that have been established by the researches of Dr. Layard. He has shown that the farther he goes back among Assyrian monuments, the more proofs accumulate of a height of civilization beyond any conceptions that had been formed from written testimony.

This much we owe to natural research, but we feel that these facts have nothing to do with any standard by which Moses is to be tried. Moses is one person, Archbishop Usher, Sir Isaac Newton and all other chronologers are different characters. That the Mosaic account of the origin of the human race is true we are well convinced. No matter how many mysterious, inexplicable facts may exist around us, which no human science can explain or penetrate, we hold that man originated just as Moses has revealed that origin. Through all the haze that surrounds every department of ethnology, through all the clouds that lour over every point, there is one bold, certain and steady light that cannot be veiled, by any art, any science nor any philosophy. No age of civilization anterior to, or coeval with Moses, no one of the intellectual powers of Assyria, Egypt, Greece or Rome ever attempted to give a solution of what, except for Moses, would have been the most inexplicable problem on earth—we mean the gift of speech. If we lay aside the Mosaic revelation on the subject, and attempt to account for this possession, we have an enigma on our hands, of more difficult solution than any known to ethnology. In all others we may grope our way, this one completely stultifies the mind. Those who have never thought upon the subject have no idea of the difficulties that present themselves in an attempt to account for the possession of the power of clothing thought with language. This point, however, is clear and unquestionable—no human being ever spake, or uttered a thought in words except by imitation. There is not an instance on record that militates against this truth. All human experience, indeed, confirms it. No one ever talked, nor will any one ever talk except from being taught language by others. No one would be dumb but for deafness, and he that never heard others speak, cannot himself speak. We have three notable instances which speak intelligibly as to what would have been the condition of the first of the human race and of all their posterity if the first pair had not been taught speech in the only way in which it cen be acquired—by hearing it. There is a young lady in this city who was able to talk up to between her third and fourth year. About that time she began to lose her hearing, and the progress of speech was checked. The defective hearing advanced until it terminated in total deafness, and in a short time she ceased to speak, because she forgot all words. Here is an instance then in which the faculty of speech existed once, while its possessor could hear, and which was completely obliterated by the loss of hearing. Some years ago, a young man, aged twenty-four, was examined by a commission of the French Academy. He had been deaf and dumb up to within four months of the time he appeared before the Academy. About four months prior to that time, he thought he felt something trickling from his ear, and upon applying his finger to it, discovered a few drops of a warm fluid. Coincident with this he heard a strange sound in his head, which he afterwards likened to the ringing of bells. The young man was overwhelmed with his sensations, but kept his newly acquired power a profound secret. He observed persons talking, and was amazed. He never had an idea on the subject until he was able to hear. During four months he kept his secret, but watched the use of speech by others in order to acquire the gift himself, and after a vigilance of four months he acquired confidence enough in his new power, to address his parents. There was certainly nothing to prevent this case from speaking, except the inability of the young man to imitate what he could not hear. The facts of this case are recorded by the French Academy after a rigid investigation of the subject.

Another remarkable case is one that occurred within the remembrances of many now living. We allude to Casper Hauser. He was born with full power of hearing, but, for reasons that have never been revealed, he was confined for nearly seventeen years to a dark hole, and his keeper never uttered a word in the boy's presence. From this imprisonment, Hauser was conveyed in a wagon, by his keeper, to Nuremberg, and turned loose in the streets, the keeper making good his retreat. A merchant of Nuremberg took charge of the boy, for the purpose of educating him, and he rapidly acquired the power of speech after he was placed where he could hear it. He lived between sixteen and seventeen years without hearing a word, and he never attempted to utter one, but as soon as he was placed where he could hear speech he acquired the gift.

These are expressive facts—they speak no dubious language. If one man cannot originate language, it is plain that one hundred could not. Men may increase the powers of a language, but this is widely different from creating one. We know what wonderful powers the imagination has and its offices, but according to John Locke and David Hume, and all other reputable metaphysicians, the imagination has not a particle of creative power. Give it an idea, or a form to work upon, and it may weave it into ten thousand forms, but it can create nothing. Thus it is with the gift of speech—man has no creative power over it—after the elements are given to him, he can extend their boundaries to a wonderful extent, but at the start, he is dependent.

These truths, which cannot be controverted, indubitably show that Moses is the only historian of the human race who ever attempted to show where and how man obtained his power of speech. If the Creator of man had not conversed with him, and thus enabled him to use his powers of imitation, the human race would have been dumb to the present hour. And the revelation of this important event shows how infinitely much more Moses knew of what he speaks in the book of Genesis, than is known among those who think themselves superior to him in more than "the learning of Egypt." In the midst of a cloud of tobacco-smoke, German philosophy teaches that Moses is a myth, but we have, in these remarks, shown that he revealed a truth, against which angry surges may beat in vain.

We yield to no one in our devotion to Ethnology in all its departments. We have cultivated it with all the power that we have been able to devote to it, and we feel the most fervent love for the whole science. But with all our love for it, we unhesitatingly declare that we would not exchange the satisfactory solution that Moses gives to the mystery of human speech for all that is known or imagined in Ethnology.

Having shown that Moses is far beyond all others in his account of the gift of speech, and that no one but the historian of the first pair on the earth ever pretended to account for the existence of that great element of human power, happiness, and progress, we think he may be trusted in other important elements of that early history. As he is rational and reliable in that important particular, and is the only one who claims any regard for the statement, he may be judged rational and reliable in those other statements where he stands alone, especially as there is nothing in the statements that are repugnant to reason.

It is of course impossible to elaborate the points we have introduced in these remarks, and our desire is rather to call the attention of medical readers to the subject of Ethnology, and to the best sources of information on that science, than to enter into a minute investigation of the various questions connected with it. The subject of Ethnology is of the profoundest importance to medical philosophers, and its elucidation must be, in a great measure, confided to them. We shall, on some future occasion, enter more fully into the merits of the questions of ethnology than we have been able to do in this number, and we shall be pleased if some of the correspondents of this Journal will give its readers an essay on the science.

Among the works named at the head of this article is Pickering's "On the Races of Man and their geographical distribution." The author, who is a physician, was a member of the scientific corps attached to the exploring expedition sent out by the Government of the United States. Dr. Pickering has made a work of great value, and deserves the thanks of all scholars for the important contributions he has made to progressive knowledge. The work is a worthy companion of Dana's Geology of the Pacific, and of Hales' Ethnography.

Dr Pickering says that he has seen eleven races of men, and is at loss, after having visited many dfferent parts of the globe, to know where to look for other races, if the eleven he has seen are not the limit. Dr. Pickering thus distributes the characteristics of the eleven races: WHITE—Arabian and Abyssinian. BROWN—Mongolian, Hottentot, and Malay. BLACKISH-BROWN—Papuan, Negrillo, Indian or Telingan, Ethiopian. BLACK—Australian, Negro.

The geographical distribution of these eleven races of men is represented on a map which accompanies the work of Dr. Pickering. It is of great value to the inquirer after ethnological science, and we commend the work as one that is indispensable in the investigation of the important subject of which it treats. The following branches of ethnological science seem to us to be handled very satisfactorily in Dr. Pickering's work: 'Associations of the races. Their numerical proportions. Relations between the races. The geographical progress of knowledge. Migrations by sea. Migrations by land. The origin of Agriculture. Zoological deductions. The introduced animals and plants of America. The introduced animals and plants of the islands of the Pacific. The introduced animals and plants of Equatorial Africa. The introduced animals and plants of Southern Arabia. The antiquities, and the introduced animals and plants of Hindostan. The Budhist caves. The Braminical caves. Domestic animals and plants of Ancient India. Introduced plants of Modern India. The introduced animals and plants of Egypt, enumerated in chronological order."

A careful study of the chapters of the work, we have thus indicated, will enable the student to place a proper value upon the claims of specific fauna and flora. Those claims have had a more conspicuous value placed upon them than they deserve, and the information imparted by Dr. Pickering forms an admirable sliding scale by which to measure the power of a number of points, that are pressed with ardor by the advocates of a diversity of origins for the races of man.

Upon the scientific department of the questions at issue, we commend Dr. Bachman's investigations. We have not had an opportunity of reading his book, but we have read the essays he has published in the Charleston Medical Journal, and those essays contain the facts, logic and principles set forth in the book itself. We have read Dr. Bachman's essays with more than ordinary satisfaction, and freely confess that they have considerably enlarged the boundaries of our knowledge.

We hope no one will so far misunderstand us as to suppose that we design in any of our remarks to use language in any degree derogatory to Professor Agassiz. We should do great injustice to our real feelings, if we were in any way to undertake to detract from his high merits. The views he has given of the races of man are those of an honest and unususally intelligent gentleman. They are supported with zeal, earnestness and power, but we have not been convinced of their correctness. There are few men, however, who may not afford to pause and reflect well before disagreeing with Professor Agassiz on any scientific subject, and we regret that we have not been able to reach the same conclusions that that eminent teacher has reached by his inquiries.

